# Novel Role of Parathyroid Hormone-Related Protein in the Pathophysiology of the Diabetic Kidney: Evidence from Experimental and Human Diabetic Nephropathy

**DOI:** 10.1155/2013/162846

**Published:** 2013-07-31

**Authors:** Montserrat Romero, Arantxa Ortega, Nuria Olea, María Isabel Arenas, Adriana Izquierdo, Jordi Bover, Pedro Esbrit, Ricardo J. Bosch

**Affiliations:** ^1^Laboratory of Renal Physiology and Experimental Nephrology, Department of Biological Systems/Physiology Unit, University of Alcalá, Alcalá de Henares, Madrid, Spain; ^2^Department of Biomedicine and Biotechnology/Cell Biology Unit, University of Alcalá, Alcalá de Henares, Madrid, Spain; ^3^Nephrology Department, Fundació Puigvert, Barcelona, Spain; ^4^Bone and Mineral Metabolism Laboratory, Instituto de Investigación Sanitaria-Fundación Jiménez Díaz, Madrid, Spain

## Abstract

Parathyroid hormone-related protein (PTHrP) and its receptor type 1 (PTH1R) are extensively expressed in the kidney, where they are able to modulate renal function. Renal PTHrP is known to be overexpressed in acute renal injury. Recently, we hypothesized that PTHrP involvement in the mechanisms of renal injury might not be limited to conditions with predominant damage of the renal tubulointerstitium and might be extended to glomerular diseases, such as diabetic nephropathy (DN). In experimental DN, the overexpression of both PTHrP and the PTH1R contributes to the development of renal hypertrophy as well as proteinuria. More recent data have shown, for the first time, that PTHrP is upregulated in the kidney from patients with DN. Collectively, animal and human studies have shown that PTHrP acts as an important mediator of diabetic renal cell hypertrophy by a mechanism which involves the modulation of cell cycle regulatory proteins and TGF-**β**1. Furthermore, angiotensin II (Ang II), a critical factor in the progression of renal injury, appears to be responsible for PTHrP upregulation in these conditions. These findings provide novel insights into the well-known protective effects of Ang II antagonists in renal diseases, paving the way for new therapeutic approaches.

## 1. Diabetic Nephropathy (DN)

End-stage renal failure due to diabetes mellitus, especially type 2 diabetes, has been recently described as a medical catastrophe of worldwide dimensions [1]. Diabetic nephropathy (DN) is characterized by the development of proteinuria and subsequent glomerulosclerosis, conditions which are always preceded by renal cell hypertrophy [2]. Although the diabetic kidney is extremely variable, from near normal size to even small fibrotic kidney, renal enlargement due to cellular hypertrophy and hyperplasia is an early feature of the disease both in human and in experimental animal models, especially in the absence of insulin treatment. Hypertrophy of tubuloepithelial as well as glomerular cells, including both visceral epithelial (podocytes) and mesangial cells, is an early hallmark of diabetes renal involvement [3–5]. Over time, glomerular cell hypertrophy might become a maladaptive response leading to glomerulosclerosis. 

Although the mechanisms by which high glucose (HG) leads to renal cell hypertrophy are still not completely understood, they appear to involve cell entry into the cell cycle—associated with cyclin D_1_ kinase activation early in G_1_—and subsequent arrest at the G_1_/S interphase, implicating inhibition or insufficient activation of cyclin E kinase, to permit progression into S phase, and therefore, arrest of cell cycle progression followed by an increase in cell protein synthesis [6].

Recent studies have shown that HG-induced hypertrophy involves an early activation of the renin angiotensin system, followed by an induction of TGF-*β*_1_, which in turn activates a cell cycle regulatory protein, the cyclin dependent kinase inhibitor (CDKI) p27^Kip1^ [7–9]. The interaction of p27^Kip1^ with the cyclin E kinase has been implicated in the inhibition of this late complex and thus the G_1_ progression [10]. 

## 2. The Renal PTH/PTHrP System

In the adult kidney, both parathyroid hormone- (PTH-) related protein (PTHrP) and the PTH1 receptor (PTH1R) are abundant throughout the renal parenchyma, including the intrarenal vasculature [11–13]. In the kidney, PTHrP appears to modulate renal plasma flow and glomerular filtration rate and induces proliferative effects on both glomerular mesangial and tubuloepithelial cells [11–17]. Renal PTHrP is overexpressed in several experimental nephropathies, including acute renal injury, obstructive nephropathy, and a rat model of tubulointerstitial scarring after protein overload, associated with the development of proteinuria [12, 18]. The recent development of a transgenic mouse model characterized by PTHrP overexpression in the renal proximal tubule made it possible to explore the functional consequences of chronic PTHrP overexpression in experimental models of renal damage (reviewed in [19]). This novel approach has provided valuable data which have helped to disclose the true roles of PTHrP in the damaged kidney. The following paragraphs describe the latest results in experimental as well as in human DN.

## 3. PTHrP in Experimental DN

Recently, we hypothesized that PTHrP involvement in the mechanisms of renal injury might not be limited to conditions with predominant damage of the renal tubulointerstitium and might be extended to glomerular diseases, such as DN. Using an experimental model of DN induced by streptozotocin (STZ) [20], we studied the possible changes in the PTHrP/PTH1R system associated with the outcome of this nephropathy, characterized by an initial phase of renal hypertrophy at both tubular and glomerular levels, followed by an increase in urinary albumin excretion (UAE) (proteinuria) [21, 22]. DN was induced in Swiss-CD1 (CD1) mice as well as in PTHrP-overexpressing mice. In the diabetic CD-1 mice, a significant increase in the expression of both PTHrP and PTH1R was observed, at both glomerular and tubular levels, associated with the development of an increase in the UAE [20]. On the other hand, diabetic PTHrP-overexpressing mice, in comparison to their control littermates, have increased renal hypertrophy, a significantly higher UAE, and lower total plasma protein levels. A significant association among the renal expression of PTHrP, PTH1R, and UAE was found to occur in the diabetic mice. Furthermore, there was a 6-fold increase in the risk of developing proteinuria in those mice with the higher PTHrP and PTH1R levels, according to the logistic regression analysis [20]. It is interesting to mention that albeit the STZ model has limitations for assessing long-term histomorphological changes in the diabetic kidney [21], the aforementioned findings might have pathophysiological implications since the amount of proteinuria is a reliable predictor of diabetic nephropathy [22]. Thus, these studies indicate that the renal PTHrP/PTH1R system is upregulated in STZ-induced diabetic mice, where it appears to be involved in renal hypertrophy and adversely affects the outcome of DN.

More recently, the putative role of PTHrP in the hypertrophy of the diabetic kidney was explored. In this way Romero et al. observed that PTHrP plays a key role in the mechanisms of HG-induced podocyte hypertrophy. It is worth mentioning that podocytes are thought to be terminally differentiated cell and hence not able to regenerate *in vivo*. In these studies, HG-induced podocyte hypertrophy was inhibited by the presence of a specific PTHrP neutralizing antibody. Interestingly, in this condition HG also failed to upregulate the expression of the hypertrophy factor TGF-*β*_1_ [23]. 

Although PTHrP does not seem to affect podocyte apoptosis, it was shown to be able to modulate the expression of several positive as well as negative cell cycle regulatory proteins. In this way, while PTHrP (1–36) was shown to stimulate cyclin D_1_, thus promoting podocytes to enter into G_1_, it also downregulates cyclin E, hence blocking the cell cycle later in G_1_. Moreover, PTHrP is able to upregulate the negative cell cycle regulatory protein p27^Kip1^ which plays a key role in diabetic cell hypertrophy by preventing activation of cyclin E activity and arresting the cell cycle later in G_1_ [4, 23]. Interestingly, Romero et al. [23] found that the pharmacological blockade of the PTH1R inhibited the p27^Kip1^ upregulation induced by both HG and AngII. Taken together, these data suggest that PTHrP might mediate the hypertrophic signaling acting in an autocrine/intracrine fashion through the PTH1R receptor. 

To discern the mechanism involved in the stimulation of p27^Kip1^ induced by both PTHrP and TGF-*β*_1_, Romero et al. [23] performed two experimental approaches. First, they found that using a PTHrP siRNA inhibited the ability of HG and AngII to stimulate the upregulation of p27^Kip1^, albeit it could not prevent the TGF-*β*_1_ upregulation of this protein. Secondly, on TGF-*β*_1_ siRNA transfected podocytes, PTHrP (1–36) failed to induce both p27^Kip1^ overexpression and hypertrophy, thus suggesting that TGF-*β*_1_ mediates both p27^Kip1^ upregulation and the hypertrophy response induced by PTHrP on HG conditions.

Interestingly, Romero et al. [23] observed that the glomerular expression of both TGF-*β*_1_ and p27^Kip1^ are constitutively upregulated in PTHrP-overexpressing mice, albeit the latter was not accompanied by renal hypertrophy [24]. This result seems plausible since the hypertrophic mechanism requires the entry into the cell cycle and subsequent arrest at the G_1_/S interphase. Several studies have demonstrated that in glomerular mesangial cells grown in HG ambient, initially, self-limited proliferation occurs due to generation of HG-induced growth factors, followed by cell cycle arrest in the G_1_ due to the expression of factors that block the checkpoint G_1_-S interphase and undergo cellular hypertrophy [4, 25–27]. Of considerable interest is the fact that previous studies on PTHrP-overexpressing mice have revealed the constitutive upregulation of various proinflammatory mediators [28], including the vascular endothelial growth factor-1 [29] without evidence of kidney damage in the absence of a renal insult. In any case, these data strongly suggest that PTHrP might participate in the upregulation of glomerular TGF-*β*_1_ and p27^Kip1^. Collectively, these results indicate that the renal PTHrP/PTH1R system is upregulated in streptozotocin-induced diabetes in mice and appears to be involved with renal hypertrophy and adversely affects the outcome of DN.

## 4. PTHrP in Human DN

In order to extend our studies into human DN, we developed two experimental approaches (30). We first assessed whether PTHrP might be upregulated in the kidney from patients with DN. And secondly, we analyzed the potential role of PTHrP in the mechanisms of HG-induced hypertrophy in another glomerular cell line known to be affected in this condition, such as human mesangial cells (HMC).

**Figure 1:**
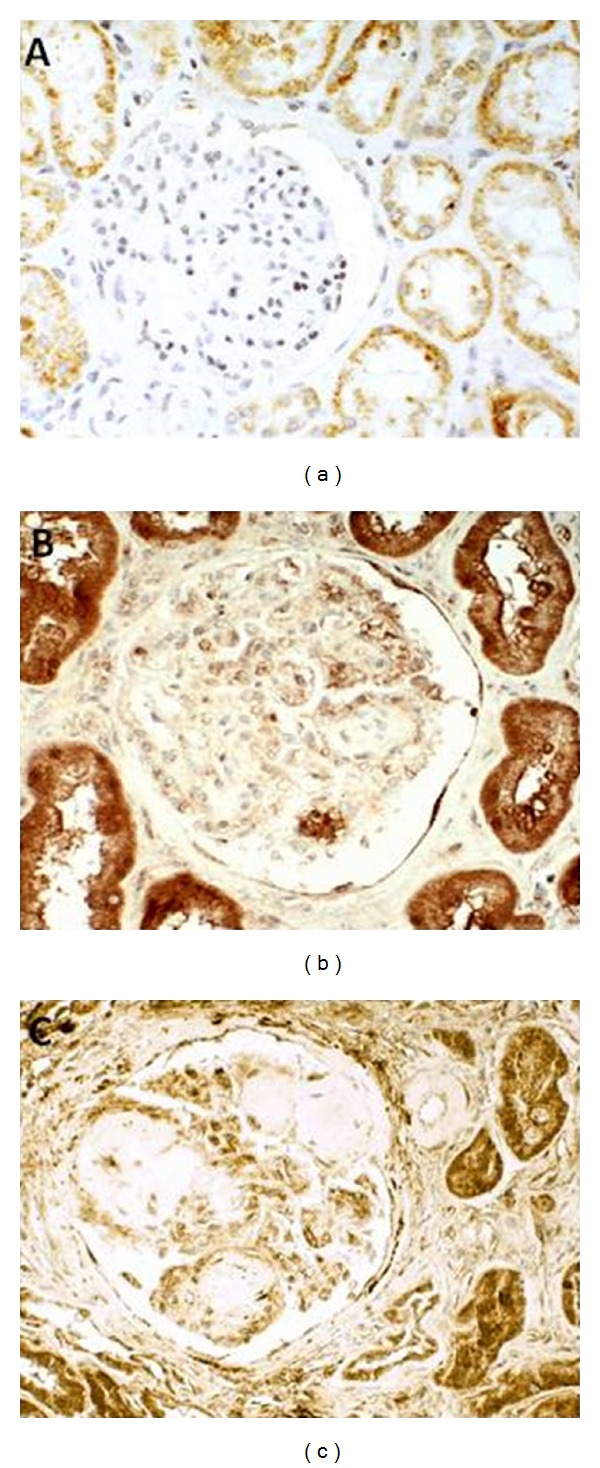
Immunostaining for PTHrP in the kidney of patients with diabetic nephropathy. PTHrP was detected by using a goat *α*-PTHrP antibody (Santa Cruz Biotechnology) in kidney tissue sections from patients with clinical and histological diagnosis of DN. (a) Section from normal kidney showing PTHrP immunostaining restricted to the epithelial cells cytoplasm of convoluted renal tubules. (b, c) Kidney samples from two diabetic patients with different degree of DN: one patient with moderate (b) or more severe (c) diabetic glomerulosclerosis. PTHrP labelling was present in both the glomeruli and the tubuloepithelial cells. Original magnifications ×300.

By using immunohistochemistry in kidney sections from patients with clinical and histopathological diagnosis of DN, we observed an intense PTHrP upregulation in both glomerular and tubuloepithelial cells, including a remarkable nuclear immunolocalization in the latter cells. Interestingly, the kidneys of these patients displayed a similar pattern of PTHrP immunolocalization to that previously observed in a diabetic mouse model [23]. Although the human diabetic kidney is extremely variable in size, renal enlargement due to hypertrophy and hyperplasia is an early feature of the disease as measured by several imaging techniques [1, 30]. Due to the fact that kidney size measurement is not regularly assessed in the clinical setting, this parameter was not available in the studied human cohort. However, the fact that all of these patients presented a pattern of PTHrP staining similar to that observed in the mouse model referred to previously, together with present *in vitro* data in HMC, strongly suggests that PTHrP may be an important factor in the pathophysiology of glomerular mesangial cell hypertrophy in diabetic patients (Figure 1).

*In vitro* studies have established that prolonged exposure of human as well as rodent MC to HG in the absence of exogenous growth factors triggers hypertrophy after a brief self-limited mitogenic effect [31]. We and other investigators previously reported that the N-terminal fragment of PTHrP is mitogenic for these cells [13, 16]. Our data herein show that HG-induced HMC hypertrophy was associated with a progressive increase in PTHrP protein expression between 24 and 72 h. Moreover, exogenous PTHrP (1–36) displays an early (24 h) proliferative effect followed by a hypertrophy response at 72 h. Thus, PTHrP seems to recapitulate the proliferative as well as the hypertrophy response induced by HG on cultured HMC (30). 

In order to study the mechanism whereby PTHrP (1–36) was able to switch its initial mitogenic stimulus into hypertrophy, we assessed the expression of several cell cycle regulatory proteins known to modulate this cellular effect. Both HG and PTHrP (1–36) were initially (24 h) shown to trigger HMC to enter the cell cycle, associated with an increase of both cyclins D1 and E and cdk2 activity. Later, at 72 h, only cyclin D1 remained increased, together with cyclin E/cdk2 inactivation. In this sense, it is well accepted that while cyclin D governs the physical growth of the cell, cyclin E determines whether the growth pattern of renal cells will be one of hyperplasia (cyclin E upregulation) or hypertrophy (cyclin E downregulation) [32]. The cdk inhibitor p27^Kip1^ is also known to play a key role in the mechanisms of HG-induced MC hypertrophy by regulating (inhibiting) the activity of the cyclin E/cdk2 complex [33, 34]. Interestingly, we also show that PTHrP (1–36) was able to upregulate p27^Kip1^ in a similar fashion and time frame as HG medium. Collectively, our findings indicate that the observed decrease in cyclin E/cdk2 complex kinase activity elicited by either HG or PTHrP (1–36) related to HMC hypertrophy is likely a consequence of both cyclin E downregulation and p27^Kip1^ upregulation. In addition, these data strongly suggest that HG and PTHrP (1–36) interact with a common cellular pathway leading to hypertrophy in HMC (30). 

The potential role of PTHrP on the mechanisms of HG-induced HMC hypertrophy was further assessed by observing that antagonizing the PTHrP system abolished the latter, together with reversal of the hypertrophy-related changes in the cell cycle (30). As we previously observed in a mouse podocyte cell line, we find that PTHrP is also able to stimulate the protein expression of TGF-*β*_1_ and its type II receptor in HMC, and a neutralizing TGF-*β*_1_ antibody abrogated HMC hypertrophy induced by PTHrP (1–36). Moreover, blockade of the PTHrP system abolished TGF-*β*_1_ upregulation but not that of its type II receptor by HG in these cells. In this regard, upregulation of the latter receptor has been shown to be associated with increased TGF-*β*-mediated growth inhibition [35], whereas its reduced expression contributes to the loss of sensitivity to TGF-*β* and the increased proliferation of some cancer cells [36, 37]. Therefore, it is likely that the TGF-*β* system might also be activated, contributing to HMC hypertrophy by a PTHrP-independent mechanism. In any event, these findings indicate that TGF-*β*1 is a downstream mediator of PTHrP (1–36) to induce hypertrophy in HMCs, as previously discussed in mouse podocytes [23]. 

## 5. Interaction between PTHrP and Angiotensin II in the Damaged Kidney

The renin-angiotensin system is well known for playing an important pathogenic role in the mechanisms of renal injury [38, 39]. Local activation of components of this system, including Ang II, in the kidney has shown to occur early in various experimental models of ARF, for example, folic acid-induced nephrotoxicity and ischemia/reperfusion [11, 39, 40]. Moreover, Ang II antagonists exert beneficial effects on renal function in these models [39, 41, 42].

Recent data strongly suggest that PTHrP might be involved in the mechanisms related to Ang II-induced renal injury. Exogenously administered Ang II, via its type 1 (AT1) receptor, increases PTHrP expression in glomerular and tubular cells as well as in vascular smooth muscle cells both *in vivo* and *in vitro* [43, 44]. Interestingly, a significant correlation between PTHrP overexpression and tubular damage and fibrosis was observed in the rat kidney after systemic Ang II infusion [43]. Furthermore, in nephrotoxic ARF, the improvement of renal function by Ang II antagonists was associated with inhibition of PTHrP overexpression [38]. These aggregated data suggest that Ang II is a likely candidate responsible for PTHrP overexpression, and this might contribute to the deleterious effects of Ang II in the damaged kidney. These findings could provide novel insights into the well-known protective effects of Ang II antagonists in renal diseases, possibly leading the way to new therapeutic approaches.

## 6. Conclusion

Collectively, these results indicate that the renal PTHrP/PTH1R system is upregulated in experimental as well human diabetes, appears to be involved with renal hypertrophy, and adversely affects the outcome of DN. PTHrP also participates in the hypertrophic signalling triggered by HG on glomerular cells. In this condition, AngII induces the upregulation of PTHrP, which might induce the expression of TGF-*β*_1_ and p27^Kip1^. These findings provide new insights into the protective effects of AngII antagonists in DN, paving the way for new forms of intervention.
